# Impact of the input of women’s working hours on household non-economic welfare

**DOI:** 10.1186/s12889-024-21063-x

**Published:** 2024-12-18

**Authors:** Liming Chu, Qi Zhang

**Affiliations:** 1 Tianjin University of Commerce, Tianjin, China; 2 Capital University of Economics and Business, Beijing, China

**Keywords:** Working hours, Household non-economic welfare, Gender differences

## Abstract

**Background:**

The economic functions of families are strengthened by the labor supply of family members. However, an in-depth discussion can still be held on the impact of the labor supply of family members on the non-economic functions of families, such as residential, social and psychological functions. This paper sought to understand the household non-economic welfare of women’s working hours. In addition, the household non-economic welfare effects of women’s working hours in terms of subdividing women’s working hours and fine-tuning household non-economic welfare were explored. The residential, social and psychological effects of women’s working hours were clarified.

**Method:**

This paper addressed the above problems based on Sen’s welfare theory and Maslow’s hierarchy of needs theory, adopted the feasible ability method, and used nationally representative, timely survey data and data from the 2014–2018 China Family Panel Studies (CFPS).

**Results:**

In this study, it was shown that the psychological effects of women’s working hours are the largest on average, with an average increase of one hour per week boosting psychological effects by 0.53%. The impact of women’s working hours on both residential and social effects has the extreme points of 68.78 and 35.89 h, respectively. By the interval, the residential effects of women’s working hours are 12.7-16.2% compared with those of weekly working hours shorter than 30 h. Additionally, women’s weekly working hours of more than 60 h have the greatest impact on residential effects, which is 16.2%. From the perspective of gender, the residential effects of women’s working hours are above those of men’s working hours. Compared to weekly working hours of less than 30 h, the social and psychological effects of women’s working hours are weaker than those of men’s working hours.

**Conclusions:**

This study not only contributes to the understanding of contributions to households from the perspective of labor value but also provides lessons for enhancing household non-economic welfare.

**Contributions to the literature:**

Limited evidence shows the contributions of working hours and healthy family development in social policies.Women’s working hours increase the residential, social and psychological effects of families. Psychological effects are the largest, with an average increase of one hour per week being associated with an increase of 0.53% in psychological effects. The impact of women’s working hours on subjective health is lower than that of men’s working hours.Few studies systematically analyze the impact of women’s working hours on family residence, socialization and subjective health, and thus target family-friendly policies.

## Introduction

Families have important welfare functions that are of great value in the survival and development of social members. The functions of families are the role they play in the development of human society and life at economic, social and psychological levels. Family development is a comprehensive process involving the replacement of generations, changes in family size, the optimization of family relationships, the enhancement of social adaptation and the improvement of the quality of family life. In this process, the economic and non-economic factors of families play an indispensable role. The family economy is the material basis for family development. In addition to economic factors, non-economic factors are also of importance in family development. The government and society have unanimously paid close attention to family development, and the relevant legal system has given active policy support to sustainable family development. Working women often find it difficult to balance their working hours with their lives and face multiple challenges. It is hard for them to find a balance due to long working hours, heavy family responsibilities and high social expectations. They are overwhelmed by the dual pressures of intense competition in the workplace and a multitude of family commitments. The economic functions of families are strengthened by the labor supply of family members. However, there is still room for an in-depth discussion of the impact of the labor supply of family members on the non-economic functions of families, such as residential, social and psychological functions.

The enhancement of family welfare reflects the value of the labor supply of family members. The Global Gender Gap Report shows that the female labor force participation rate of China was 63.73% in 2023, which ranked first among the major economies worldwide and far exceeded the international average of 47.29%. Labor input is the basis for maintaining family survival. Existing research shows that labor supply is one of the most important sources for improving the economic resources of families [1, 2]. Despite all the attention paid by current research to topics related to the family welfare effects of individual labor supply, only a small number of studies have systematically analyzed the influence of women’s working hours on the level of household non-economic welfare. In terms of research objects, studies focusing on the contributions of women’s labor value to families are insufficient. Professional women are a group of multidisciplinary concerns. Nevertheless, little research focuses specifically on the impact of women’s working hours on the level of household non-economic welfare. Regarding research methodology, empirical studies on the household non-economic welfare of women’s working hours and the mechanisms behind women’s working hours on family development, such as family security, society and self-value demonstration, are inadequate [3].Concerning research perspectives, only a small amount of research links occupational working hours to household welfare. Existing studies predominantly focus on the welfare of agricultural households, the factors affecting working hours and household economic welfare. However, the family effects of women’s working hours, especially household non-economic welfare, are mostly neglected.

This paper addressed the above problems based on Sen’s welfare theory and Maslow’s hierarchy of needs theory, adopted the feasible ability method and utilized nationally representative and timely survey data. The focus was on the household non-economic welfare of women’s working hours. The household non-economic welfare effects of women’s working hours in terms of subdividing women’s working hours and fine-tuning household non-economic welfare were explored. The residential, social and psychological effects of women’s working hours were clarified. Based on the empirical analysis results, the pulse of countermeasure suggestions to improve family welfare was taken. The effects of the investment of women’s working hours on household non-economic welfare were investigated from the angles of subdividing women’s working hours and refining household non-economic welfare. The residential, social and psychological effects of the investment of women’s working hours were clarified. Then, recommendations were made based on the empirical analysis results to enhance family welfare. Employment is the foundation of female survival and development. The labor supply of women is associated with not only their own quality and economic status but also family factors, which are important for the stability and development of the whole society. Revealing the family effects of the investment of women’s working hours can help to enrich the theoretical framework and practical reference of existing research. In theory, it is beneficial to make up for the academic research on the family effects of working hours. In policy terms, it will refine household non-economic welfare and clarify the effects of women’s working hours on the household living environment, family social capital and subjective psychological state. This will provide scientific data support for the policies of the government to improve the system of working hours and work arrangements and promote sustainable family development.

## Literature review

Family welfare is the effect of the interactions between various subjects of interest like society and the government. The understanding of family welfare by the community is mostly based on the working practice and experience of the government rather than the original intention of family welfare, which combines its theory and practice. Family welfare generally refers to the behaviors and measures in the areas of life, education, healthcare, etc., which are shared by the government or society to undertake or enhance the functions of families [4]. Through the summary of existing studies, it can be seen that family welfare can include several aspects such as social security status, family economic status, family living conditions, community life, living environment and psychological status [5–7]. Notwithstanding the wide range of connotations of family welfare, it can be summarized that family welfare can be measured by two levels of content and two dimensions of economic and non-economic conditions according to different research topics and objectives [8, 9]. Family functioning encompasses the structural and social attributes of the family environment, with key attributes including family communication, organization, roles, cohesion, conflict, emotional and behavioral responses, and problem-solving abilities. It is a measure of the functioning state of the family system. Family welfare is understood in terms of family functioning. That is to say, families provide material security and psychological and social support for individual survival and development.

As regards household non-economic welfare, its connotation is uncertain, and its measurement is difficult to standardize. Research is mostly nested in family welfare. Based on the viability theoretical framework of Sen, it can be found from the research on related connectivity that family welfare includes environmental conditions, social participation and support, social security, children’s education and social equity in addition to the family economic situation [10]. Based on different research focuses and methods, the commonality of household non-economic welfare lies in reflecting the hope, value and happiness of families. Related studies have focused on household economic welfare and comprehensive family welfare measurement [11], and few have centered specifically on their non-economic welfare. The influencing factors of household economic welfare can be summarized as macro and micro factors. The former includes food price volatility, agricultural insurance, institutional change, local decentralization, wage systems and labor mobility [12], and the latter contains educational, gender differences, labor force employment and health [13–16]. Some scholars have also argued that the well-being of families is affected by changes in health status, such as height, body mass index, health care status, nutritional intake and diseases, all of which have an impact on family welfare [17]. With the deepening of population aging, the impact of health risks will reduce the number of labor force participants. Thus, this reduces the labor participation time of labor force participation, which will adversely affect the improvement of social welfare and national economic development of Chinese residents [18].

From the perspectives of working hours and household non-economic welfare, a literature search using the term “household non-economic welfare” shows that only a handful of studies have mentioned the impact of working hours on household non-economic welfare. A majority of relevant studies have been scattered in the analysis of the connotations of family welfare. As for the effects of working hours on the household living environment and subjective psychological state, living conditions and areas are the basis for ensuring family life, clothing, food, housing, transportation and other activities. The better the living conditions are, the larger the living areas will be, which not only better meets the basic needs of life and ensures the independence of family members’ space and privacy, but also guarantees many extended functions such as learning, fitness and leisure. Labor force participation in the labor market improves family economic income, family living conditions and the household living environment on the one hand, and worsens the living environment due to the reduction in the input of housework time on the other hand [19]. As a rule, family members increase their working hours for a better life. Increasing working hours brings obvious pressure on resident families to a certain extent, which will reduce their welfare levels. Wage income and social status are relatively low, which cannot meet the diversified consumption needs and psychological demands of resident families [20]. In their exploration of the impact of family welfare on farming households, scholars have concentrated on the raising and caring of children and the elderly in families. Supporting the elderly, whether in time investment, material consumption or social and family support, can be seen as a resource for intergenerational support [21]. Therefore, scholars have argued that caring for the elderly by lowering market input or providing social services can boost the well-being of families. In general, women serve as both family caregivers and social workers, and their work behaviors also exert an impact on family input time and welfare [22]. Although the pressure of child rearing has gradually leveled off, the child rearing model will gradually shift to a centralized rearing model with the scarcity of labor and working hours. This phenomenon has already occurred in modern society. It can be said that “outsourcing” child-rearing enables women to arrange more time for work, which brings the greatest benefit to families. It is foreseeable that labor costs and work remuneration will increase in the long term with the change of labor resources from surplus to scarcity, which will lead to a greater tendency for the labor force to concentrate on a centralized mode of allocating personal time. In this way, family members can devote more time and energy to market economic activities [23].

Overall, the current research studying the impact of women’s working hours on household non-economic welfare has a number of shortcomings. This is mainly because most of the existing research zooms in on the indicators and measures of family welfare, and very little of it is devoted to studying household non-economic welfare. Studies have shown that increasing women’s working hours is conducive to improving household economic welfare, and families also provide material, psychological and social support for individual survival and development. From this point of view, women’s working hours positively affect the social and psychological dimensions of families. However, this inference needs to be supported by the results of empirical analyses. Meanwhile, existing research mainly zeros in on the group of farming households and seldom probes into the household effects of women’s working hours. More than that, most literature has not examined in depth the impact of the investment of working hours on household non-economic welfare from a gender perspective.

## Theoretical hypotheses

According to household non-financial welfare from Maslow’s hierarchy of needs theory and Sen’s viability theory, Maslow’s hierarchy of needs theory classifies human needs into five levels from low to high: physiologicalneeds, safetyneeds, love and belongingness, esteem, and self-actualization [24]. These hierarchies of needs apply to both individuals and families. Following Maslow’s hierarchy of needs theory, household non-economic welfare is mainly reflected in the satisfaction of the social, respect and self-actualization needs of family members. The fulfillment of these needs enhances family members’ sense of well-being, contentment and belonging. As a result, the overall well-being of families is enhanced. Sen’s viability theory emphasizes the important role of human freedom and capabilities in achieving well-being [25]. In the light of Sen’s viability theory, a favorable living environment in a family means that family members can enjoy a better quality of life and a higher degree of freedom in their life [26]. The internal relationships within families and external social relationships constitute the family support network, which has a profound impact on the life quality of families. Family members’ attitudes towards life and their emotional needs and psychological well-being are important factors influencing household non-economic welfare.

From academic research and policy guidelines, this thesis defined household non-economic welfare as family welfare that does not directly involve material wealth, but significantly influences the well-being and life quality of family members. According to this definition, household non-economic welfare helps family members to adapt to society at the non-material level, improves the happiness and satisfaction of family life, and promotes the healthy and sound development of family members’ personalities through writing or external means among family members.Based on the above analysis, the relationship between women’s working hours and household non-economic welfare was analyzed from three aspects, namely the household living environment, family social relations and subjective psychological state.

The family living environment is a comprehensive reflection of housing conditions and quality. From the family perspective, housing is one of the most basic living conditions for people’s survival and development, and also has the functions of counteracting economic difficulties and providing economic security, which will increase the self-confidence and sense of individual security of the population. Living conditions improve with the increase of working hours and the rise of people’s living standards. Hence, the relationship between women’s working hours and the household living environment is manifested in the fact that an increase in working hours helps to improve the living conditions and quality of households, which in turn enhances the household living environment. Economic income becomes the main factor influencing living patterns. With the investment of working hours, the comfort, safety and tidiness of living will be enhanced through the use of market services and smart homes, which in turn will enhance the household living environment. Nonetheless, the quality of living cannot be entirely dependent on the use of smart homes and household appliances. Increasing working hours, i.e., prolonging the investment of an individual in the labor market, can usually directly enhance the economic income level of a household. This economic gain undoubtedly enhances the ability of households to meet their basic living needs and pursue a higher quality of living space. For example, households may be able to move into more spacious homes, improve their living conditions or provide better educational environments for their children, all of which directly embody the increased satisfaction of their needs for living space. Working hours can improve the ability of a family to meet its domestic living space needs, but may negatively affect life quality. Under the circumstances, outsourcing household chores is probably an option despite not being a complete one. Domestic work is mainly undertaken by women, and the commitment of women’s working hours can partly enhance the ability of households to purchase a home and satisfy the demand for objective conditions like living space for families. Subject to time commitment constraints, women may not have enough time and energy to maintain and improve the living environment, like carrying out household cleaning, greening and landscaping. This will not only reduce the comfort of the living environment but may also affect the living experience and sense of well-being of family members, which will negatively affect potential needs like the living space quality of families. Thus, women’s working hours can enhance the ability of families to buy a house and meet the objective demand for household living space but will have a negative impact on the potential demand for the living quality of households. Therefore, working hours may have a rising and then declining impact on the household living environment. Compared with short working hours, different degrees of working hours will exert a stage-by-stage impact on household living conditions and quality, which in turn affects the household living environment. For this reason, women’s working hours have different degrees of impact on the household living environment. Accordingly, the first hypothesis of this paper was formulated:

### H1a:

In terms of the impact trend, women’s working hours positively affect the household living environment.

### H1b:

In terms of the impact relationship, an inverted U-shaped relationship can be found between women’s working hours and the household living environment.

### H1c:

In terms of the impact degree, a stage-wise relationship can be observed between the commitment of women’s working hours and the household living environment.

From the perspectives of working hours and family social relations, family social relations expand and strengthen social networks by influencing social interactions on the one hand, and maintain kinship relations and help families to obtain more opportunities and resources on the other hand. The increase in women’s working hours may expand reciprocity and visits between family members and friends, continue the relationship between family members and friends, increase the frequency of social exchanges and communication, and deepen friendship exchanges. Hence, the impact of women’s working hours on family social relations is embodied in the following way: The increase in working hours contributes to accumulating personal connections and enhancing identity status, which brings about more social support and networks and thus strengthens family social relations. Family social relations are primarily reflected in the accumulation and broadening of human resources, families and friend relations, all of which require a certain amount of time investment. Women’s level of social support is limited when the investment of women’s working hours is insufficient and women lack social networks with colleagues and leaders, as well as exchanges with neighbors, friends and relatives. Consequently, family social relations may decline, and the level of social relations rises with the increasing investment of women’s working hours after the accumulation of social resources to some degree. Therefore, the input of working hours has a downward and then upward impact on the economic status of families. The social rights and resources gained from the investment of working hours in turn affect the changes in family social relations, and the stage-by-stage effects of the investment of women’s working hours may be weaker. Hence, different levels of investment of working hours may have no significant effect on family social relations. Accordingly, the second hypothesis of the paper was formulated:

### H2a:

In terms of the impact trend, the investment of women’s working hours positively affects family social relations.

### H2b:

In terms of the impact relationship, a U-shaped relationship can be found between women’s working hours and family social relations.

### H2c:

In terms of the impact degree, no stage-wise relationship can be observed between the investment of women’s working hours and family social relations.

From the viewpoints of working hours and subjective psychological state, work is an important criterion for measuring personal value and an important guarantee for a happy life. Women who increase their input of working hours can gain a sense of satisfaction and achievement from their work. Therefore, the association between the investment of women’s working hours and subjective psychological state is manifested in the fact that the investment of women’s working hours positively affects subjective psychological state. Due to the subjectivity of the psychological state, insufficient and excessive working hours can be analyzed from different theories and perspectives to draw different conclusions. The analysis of the resource consumption theory demonstrates that insufficient working hours can be viewed as resource depletion and marginal effects are reduced, which in turn will affect the subjective psychological state. When women’s working hours are insufficient or fail to reach the desired working hours, workers will think that resource acquisition is insufficient, which will reduce their subjective psychological state. From the perspective of preferences for leisure, shorter working hours will enhance the subjective psychological state when workers prefer leisure. The analysis of the resource consumption theory reveals that too many working hours will adversely affect workers’ physical and mental health and even family relationships. The length of working hours is also linked to income, which affects the objective quality of life and brings about a positive subjective psychological state. Thus, working hours can have an upward and then downward or downward and then upward effect on a subjective psychological state. The positive emotional experience and the negative effects of ego depletion brought by working hours will have a stage-by-stage effect on the subjective psychological state in comparison with that brought by rigid working hours. As a result, different levels of working hours may have no significant effect on subjective psychological state. Accordingly, the third hypothesis was proposed:

### H3a:

In terms of the impact trend, the commitment of women’s working hours positively affects subjective psychological state.

### H3b:

In terms of the influence relationship, a U-shaped or inverted U-shaped relationship can be found between the commitment of women’s working hours and subjective psychological state.

### H3c:

In terms of the impact degree, a stage-wise relationship can be observed between the commitment of women’s working hours and subjective psychological state.

## Methodology, variables, and data

### Data sources

Data from the China Family Panel Studies (CFPS) of Peking University in 2014, 2016 and 2018 were used for empirical analysis in this paper. The research sample was based on the working age of the labor force. Given the arrival of the aging society of China, the labor force has reached the retirement age but “not retired”. According to the research theme of this paper, the value of working hours is free from the influence of the retirement policy. Based on the above considerations, the sample of the female non-agricultural labor force was relaxed and restricted to the 16–64 age group of the labor force. Non-agricultural employment was defined as non-agricultural employment if the respondents answered “have a job” and “work in agriculture” with “no”. In the sample of gender comparison, the labor force in the male non-agricultural labor force sample was aged 16–64. In this paper, the personal, household and family welfare data in the female labor force sample were combined. The information on women’s working hours and personal and occupational characteristics was mainly obtained from the adult questionnaire data of the CFPS, and the indicators of household characteristics and non-economic welfare were from the household and adult questionnaire data of the CFPS.

### Variable selection and description

#### Main variables

Dependent variables.Household non-economic welfare variables, were measured by the following indicators, including the household living environment, family social relations and subjective psychological state, with reference to existing studies in the academic world and relevant information in databases.

Indicator of the household living environment (fhouseb): A composite indicator of the number of homes owned and the degree of tidiness of homes was chosen as a proxy variable for measurement. The household living environment reflects the living needs and comfort level of houses. The larger the value is, the higher the comfort level will be. The indicator positively affects the realization of the living function. The number of houses owned and the degree of cleanliness of homes represent the living conditions and quality of households. They were measured by the responses to the questions “Who owns the current house of your family”, “Do you have any other property besides the current house” and “What is the degree of cleanliness of the home”. The responses to the questions “Who owns your current home?”, “Do you have a property other than your current home?” and “What is the degree of tidiness of your home?” were selected to measure the quality of household living conditions.

Indicator of family social relations (lnscexp): Expenditure on favors and gifts was chosen as a proxy variable for measurement [27]. The existing research in literature and the characteristics of the CFPS database were taken into consideration, and the expenditure on favors was used to measure family social relations. The responses to the sample questionnaire, “Including both in-kind and cash, household expenditure on gifts of favors in the past 12 months”, were selected as the measure.

Indicators of subjective psychological state (Happiness): Happiness was chosen as a proxy variable for measurement. The question “How happy (score)” in the sample questionnaire was selected as a proxy for subjective psychological state based on the respondents’ subjective feelings of happiness.

Independent variable. Working hours were selected as the core explanatory variable. The measure used by academics in the study of working hours is generally weekly working hours as a measure. In the meantime, working hours were measured in three dimensions: The first one was the continuous variable of working hours; the second one was the inclusion of the squared term of working hours; the third one was the division of working hours into the five time periods of (1,30], (30,40], (40,50], (50,60] and (60,max] hours. The measurement of working hours at these three levels comprehensively presented their impact on household non-economic welfare in the aspects of the trend, extent and specific relationship of working hours. The three dimensions of working hours were considered in different models.

**Table 1:** Descriptive statistics of core variables

		*N*	Max	Min	Mean	SD	p50
Men							
	Fhouseb	13,350	6.00	1.00	4.76	1.09	5.00
	Lnscexp	13,973	10.31	1.61	7.87	1.11	8.01
	Hap	10,809	10.00	0.00	7.60	2.04	8.00
	Worktime	15,645	105.00	6.00	53.41	18.41	56.00
Women							
	Fhouseb	8610	6.00	1.00	4.73	1.14	5.00
	Lnscexp	9283	10.31	1.61	7.92	1.11	8.01
	Hap	7143	10.00	0.00	7.79	2.01	8.00
	Worktime	10,308	105.00	6.00	51.09	18.57	50.00

Table 1 shows the descriptive statistics of core variables. The average values of working hours were 51.09 and 53.41 for women and men, respectively. Comparative analyses revealed that the mean value of the household living environment was higher for the male sample at 4.76 than for the female sample, and the mean values of family social relations and subjective psychological status in the male sample were lower than those of the female sample.

#### Control variables

The impact of women’s working hours on the household living environment, family social relations and subjective psychological state was examined in this paper. Considering the rationality of the model in terms of its economic implications, different dependent variables called for the introduction of different control variables. Based on the research theme, the control variables of household non-economic welfare were divided into three main categories: personal, work and family characteristics. The first category is personal characteristics, including age, urban household registration or not, marital status, education level and health status. The second category is work characteristics, including personal income and unit nature. The third category is family characteristics, including household size, the number of young children and old people, and time spent on housework. To control for differences between regional levels of economic development, gross domestic product (GDP) per capita in the province or city of individuals was introduced, and occupation, region, and year fixed effects were added.

### Model construction

Dependent variables must be continuous variables according to the requirement of the ordinary least squares (OLS) model, and below is the specific measurement model:

1 $$\:{\text{Y}}_{1i}={a}_{0}+{a}_{1}{worktime}_{i}+\alpha\:{X}_{i}+{\vartheta\:}_{i}+{\gamma\:}_{i}+{\delta\:}_{i}+{\epsilon\:}_{i}$$

Where $\:{\text{Y}}_{1i}\:$is the dependent variables; $\:{worktime}_{i}$ is the independent variable that represents working hours and is measured using the number of weekly working hours;$\:{X}_{i}$ stands for the corresponding vector of control variables;$\:{\vartheta\:}_{i}$, $\:{\gamma\:}_{i}$, $\:{\delta\:}_{i}$ indicate occupation, region and year fixed effects, respectively; $\:{\epsilon\:}_{i}$ refers to the random disturbance term.

The ordered probit (Oprobit) model treats dependent variables as ordinal variables and requires using latent variables to derive maximum likelihood estimation (MLE) estimates.

2 $$\:{Y}_{2i}^{*}={b}_{0}+{b}_{1}{worktime}_{i}+\beta\:{X}_{i}+{\vartheta\:}_{i}+{\gamma\:}_{i}+{\delta\:}_{i}+{\epsilon\:}_{i}$$

where$\:\:{Y}_{2i}^{*}$ denote the i^th^ dependent variables, with which $\:{\text{Y}}_{2i}\:$has a certain quantitative relationship. When $\:{Y}_{2i}^{*}$ is lower than the critical value $\:{C}_{1}$, the dependent variables is the minimum value ($\:{\text{Y}}_{2i}=1$). When $\:{Y}_{2i}^{*}$ is above $\:{C}_{1}$ but below the critical value $\:{C}_{2}$, the dependent variables is larger ($\:{\text{Y}}_{2i}=2$). When $\:{Y}_{2i}^{*}$ is higher than $\:\:{C}_{6}$, the dependent variables is at its maximum value ($\:{\text{Y}}_{2i}=6$). As shown in Eq. (3):

3 $${{\rm{Y}}_{2i}} = \left\{ {\matrix{1 & {Y_{2i}^* \le {C_1}} \cr 2 & {{C_1} < Y_{2i}^* \le {C_2}} \cr \vdots & \vdots \cr {\rm{m}} & {Y_{2i}^* > {C_{j - 1}}} \cr } } \right.$$

Assuming ε_ijk_∼(0,1) distribution, X denotes all independent variables, and Φ(·) represents the cumulative distribution function. $\:{\text{Y}}_{2i}$ can be expressed as:

4 $$\:\text{P}\left({\text{Y}}_{2i}=1\right)={\Phi\:}({C}_{1}-\text{X}{\upbeta\:})$$

5 $$\:\text{P}\left({\text{Y}}_{2i}=2\right)={\Phi\:}\left({C}_{2}-\text{X}{\upbeta\:}\right)-{\Phi\:}({C}_{1}-\text{X}{\upbeta\:})$$

6 $$\:\text{P}\left({\text{Y}}_{2i}=6\right)=1-{\Phi\:}\left({C}_{6}-\text{X}{\upbeta\:}\right)$$

Where $\:{\text{Y}}_{2i}\:$ represents dependent variable; $\:worktime$ stands for the independent variable that denotes working hours and is measured by the number of weekly working hours; $\:{X}_{i}\:$indicates the corresponding sets of control variables; $\:{\vartheta\:}_{i}$, $\:{\gamma\:}_{i}$, $\:{\delta\:}_{i}\:$denote occupation, region and year fixed effects, respectively; $\:{\epsilon\:}_{i}\:$is the random perturbation term.

To analyze the association of the impact of women’s working hours with household non-economic welfare, the squared term of working hours (worktime2) is introduced into Eqs. (1) and (2), respectively. To analyze the impact degree of women’s working hours on household non-economic welfare, working hours are replaced by working hours in the sub-intervals of Eqs. (1) and (2), respectively to carry out the empirical analysis.

## Results and discussion

### Impact of the commitment of women’s working hours on the household living environment

The impact of the investment of women’s working hours on families can be seen through changes in the household living environment. Housing is an important component of family welfare. The household living environment can be used to determine the housing needs, fixed assets and income of families in some measure. It is used as a basis for assessing the effect of the investment of women’s working hours. A composite indicator of the number of houses owned and the degree of cleanliness of homes was chosen as a measure of the living environment.

Based on existing research, the household living environment is normally associated with the financial liabilities borne by households and the value of the present housing stock. Concerning the economic attributes of the household living environment, both financial liabilities and the value of the present housing stock affect the number of owner-occupied dwellings owned by households. Therefore, financial liabilities and the value of the present housing stock were included as control variables when the household living environment was used as an dependent variable.

The regression results of women’s working hours on the household living environment are illustrated in Table 2. The effects of the duration of women’s working hours, the squared term of working hours and varying degrees of working hours on the household living environment are presented in columns (1)-(9). The estimation results of the Oprobit model for the main analysis are reported in columns (1)-(6), and the estimation results of the OLS model as a robustness test are demonstrated in columns (7)-(9).

According to Table 2:

First, the increased input of women’s working hours contributes to the improvement of the household living environment. Observations show that women’s working hours have significantly positive regression coefficients in both the Oprobit model with the family social environment as an ordinal variable and the OLS model regression considering it as a continuous variable. This indicates that women’s working hours are conducive to optimizing the household living environment. Independent variables significantly enhance the household living environment under different estimation methods. In terms of women’s working hours, each increase of one hour in weekly working hours enhances the household living environment by 0.15, which is 3.17% of the mean value of the household living environment (4.73). The living environment is a factor in household non-economic welfare. The demand for living conditions is increasingly focused on the visual experience and comfort of housing in addition to the need for survival and living conditions. More time is devoted to market work to satisfy the need to improve family housing conditions and the household living environment.

Second, the association of women’s working hours and the household living environment is an inverted “U” shape, with a phased effect. Through calculation, it can be seen that women’s weekly working hours reach the inflection point of about 69 h and begin to have an inhibitory effect on the household living environment. Combined with the average value of women’s working hours, it can be preliminarily determined that women’s working hours at this stage have a positively impact on household living environment. Weekly working hours shorter than 30 h were taken as the reference group. In addition to 40–50 h, the three groups of 30–40 h, 50–60 h and more than 60 h of weekly working hours all increase the degree of influence on the household living environment. In particular, the optimization of the household living environment is more than 15% when working hours are in the range of 50–60 and more than 60 h. The hypotheses proposed in this study were all verified. The possible reason for the negative impact of the investment of women’s working hours on the household living environment is as follows: The investment of working hours up to a certain level, which may satisfy the economic attributes of the household living environment, is unable to satisfy environmental attributes due to the reduction of the time investment allocated to households. This results in an overall downward trend in the household living environment.

Third, age, education level, personal income, type of workplace, family size and current housing value all have a bearing on the household living environment. Established studies have also confirmed that the demand for improvements in the household living environment increases with age. Higher education levels and income will enhance purchasing power and accordingly increase the awareness of home ownership. The effect of housing provident fund influence varies by the type of workplace, which in turn may have different impacts on the household living environment. Changes in the household living environment are generally based on households as a unit. By and large, the demand for the housing space and quantity of households will increase when the household size is larger. Current housing value mirrors the demand of households for the living environment. Hence, household size and current housing value have a positive correlation with the demand of households for housing. The reason why the impact of household saving rate is not significant on the household living environment may be that the effect of savings on the household living environment is twofold. In terms of stock savings, savings have a positive wealth effect on households. That is, capital accumulation will propel residents to buy a home. From the point of view of incremental savings, savings have a substitution effect on households. In other words, the household saving rate will fall if households choose to purchase a home.

**Table 2:** Impact of women’s working hours on the family’s living environment

	(1)	(2)	(3)	(4)	(5)	(6)	(7)	(8)	(9)
	Oprobit						Robustness tests: replacing the model		
Worktime	0.00146^*^	0.00151^*^	0.00478^*^	0.00642^**^			0.00130^*^	0.00546^**^	
	(0.087)	(0.079)	(0.088)	(0.028)			(0.076)	(0.028)	
Worktime2			-0.0000594^**^	-0.0000467^*^				-0.0000396^*^	
			(0.022)	(0.075)				(0.077)	
Working time (Less than 30 h = reference group)									
(30,40]					0.213^***^	0.127^**^			0.112^**^
					(0.000)	(0.017)			(0.013)
(40,50]					0.117^**^	0.0742			0.0666
					(0.025)	(0.169)			(0.146)
(50,60]					0.158^***^	0.162^***^			0.135^***^
					(0.004)	(0.003)			(0.004)
(60,max)					0.154^***^	0.162^***^			0.136^***^
					(0.005)	(0.003)			(0.004)
Age		0.00447^***^		0.00464^***^		0.00458^***^	0.00402^***^	0.00418^***^	0.00412^***^
		(0.002)		(0.001)		(0.002)	(0.001)	(0.001)	(0.001)
Educ		0.0743^***^		0.0702^***^		0.0725^***^	0.0646^***^	0.0611^***^	0.0625^***^
		(0.000)		(0.000)		(0.000)	(0.000)	(0.000)	(0.000)
Lnwage		0.0597^***^		0.0641^***^		0.0661^***^	0.0492^***^	0.0529^***^	0.0542^***^
		(0.000)		(0.000)		(0.000)	(0.001)	(0.000)	(0.000)
Workplace		0.121^***^		0.119^***^		0.115^***^	0.0940^***^	0.0923^***^	0.0877^***^
		(0.001)		(0.002)		(0.003)	(0.003)	(0.003)	(0.006)
Familysize		0.0540^***^		0.0542^***^		0.0547^***^	0.0503^***^	0.0504^***^	0.0508^***^
		(0.000)		(0.000)		(0.000)	(0.000)	(0.000)	(0.000)
Number of children		-0.0188		-0.0185		-0.0188	-0.0158	-0.0156	-0.0158
		(0.347)		(0.355)		(0.348)	(0.355)	(0.363)	(0.357)
Housework		-0.00864		-0.00789		-0.00699	-0.00629	-0.00568	-0.00493
		(0.409)		(0.452)		(0.505)	(0.482)	(0.525)	(0.581)
Financial liabilities		-0.00526		-0.00504		-0.00512	-0.00539^*^	-0.00519^*^	-0.00525^*^
		(0.143)		(0.162)		(0.154)	(0.082)	(0.094)	(0.090)
Household saving rate		-0.0107		-0.0123		-0.0124	-0.000737	-0.00200	-0.00198
		(0.671)		(0.628)		(0.624)	(0.972)	(0.924)	(0.924)
Housing value		0.111^***^		0.110^***^		0.110^***^	0.0902^***^	0.0894^***^	0.0892^***^
		(0.000)		(0.000)		(0.000)	(0.000)	(0.000)	(0.000)
GDP per capita		-0.0833		-0.0837		-0.0783	-0.0751	-0.0755	-0.0720
		(0.179)		(0.177)		(0.208)	(0.148)	(0.146)	(0.166)
Occupational fixed effects; Regional fixed effects; Year fixed effects		Yes		Yes		Yes	Yes	Yes	Yes
Constants							5.248^***^	5.158^***^	5.171^***^
							(0.000)	(0.000)	(0.000)
*N*	6447	6447	6447	6447	6447	6447	6447	6447	6447
*Log likelihood*	-7998.2592	-7937.6946	-8025.4703	-7936.1541	-8032.1887	-7932.8613			
*R* ^2^							0.407	0.407	0.408
F							300.3	288.1	266.4

Note *, ** and *** indicate significant at the 10%, 5% and 1% levels respectively, with p-values reported in parentheses

### Impact of the commitment of women’s working hours on family social relations

Based on existing research, whether urban household registration, health status, marital status, party membership, type of work unit, and number of elderly people can reflect the differences between urban and rural areas, health, family responsibilities, and social resources, and more accurately reveal the complex links between working hours and family social relations, thus the above variables are added to the regression model.

The impact of women’s working hours on family social relations are shown in Table 3 were based on OLS regressions. The effects of the length of women’s working hours, the squared term of working hours and different levels of working hours on family social relations are displayed in columns (1)-(6).

According to Table 3:

First, the increase in the commitment of women’s working hours promotes family social relations. Regarding the significance of the coefficient on women’s working hours, the regression results show that women’s working hours have a significantly positive coefficient. Specifically, an increase of one hour in women’s weekly working hours is associated with an average increase of 0.27% in family social relations. An increase in women’s working hours can enhance women’s self-confidence and integration into social groups and the continuous building of good interpersonal relationships, which is important for family survival and development. When entering the labor market and cooperating and communicating with social groups or colleagues, women have to establish certain interpersonal relationships with others for their survival and development. Increased working hours mean more time devoted to the labor market and communication with others. It is also a way of maintaining social networks, bringing social resources to families and thus enhancing family social relations.

Second, women’s working hours have a “U”-shape impact on family social relations, with no phase effects. In terms of the relationship between the two, the primary and secondary terms for women’s working hours are negative and positive, respectively. Statistically, the relationship between the two is in the shape of a “U” curve. Calculations reveal that women’s weekly working hours begin to positively affect family social relations when reaching an inflection point of approximately 36 h weekly. With the reference group of less than 30 h weekly, the impact of working hours on family social relations is not sensitive at all intervals. Hypothesis 3 was fully verified. The possible reason for the lack of a phase-wise effect of women’s working hours on family social relations is that maintaining social relations and broadening the size of social networks typically require a cumulative process, and inter-area differences in working hours have no direct impact on the changes in social relations. Thus, different levels of working hours may not have a significant effect on family social relations.

Third, health status, marital status, personal income and state-owned enterprise units show a significant positive correlation with family social relations. In general, better health status, married status, higher personal income and working in state-owned enterprises may contribute to broadening family social relations. The number of children and elderly people and household saving rate exhibit a significant negative correlation with family social relations. A high number of children and elderly people in a household and the tendency of a household to save may inhibit the social interactions of family members.

**Table 3:** Impact of women’s working hours on family social relations

	(1)	(2)	(3)	(4)	(5)	(6)
Worktime	0.00134^*^	0.00272^***^	-0.0113^***^	-0.00585^**^		
	(0.081)	(0.001)	(0.000)	(0.036)		
Worktime2			0.0000790^***^	0.0000815^***^		
			(0.000)	(0.001)		
Work time(Less than 30 h = reference group)						
(30,40]					0.00368	0.0314
					(0.931)	(0.538)
(40,50]					-0.124^***^	-0.0659
					(0.004)	(0.201)
(50,60]					-0.243^***^	-0.00922
					(0.000)	(0.860)
(60,max)					-0.172^***^	0.0742
					(0.000)	(0.163)
Age		0.00467^**^		0.00431^**^		0.00445^**^
		(0.020)		(0.032)		(0.027)
Register		-0.0457		-0.0422		-0.0443
		(0.133)		(0.164)		(0.145)
Educ		0.0803^***^		0.0873^***^		0.0829^***^
		(0.000)		(0.000)		(0.000)
Health		0.0224^*^		0.0231^*^		0.0200
		(0.065)		(0.057)		(0.101)
Marriage		0.233^***^		0.234^***^		0.233^***^
		(0.000)		(0.000)		(0.000)
Member		0.263^***^		0.267^***^		0.260^***^
		(0.003)		(0.002)		(0.003)
Lnwage		0.166^***^		0.161^***^		0.153^***^
		(0.000)		(0.000)		(0.000)
Workplace		0.116^***^		0.117^***^		0.0983^***^
		(0.001)		(0.001)		(0.005)
Number of children		-0.173^***^		-0.171^***^		-0.172^***^
		(0.000)		(0.000)		(0.000)
Number of old		-0.0664^***^		-0.0654^***^		-0.0692^***^
		(0.002)		(0.002)		(0.001)
Housework		0.00280		0.00184		0.00181
		(0.791)		(0.861)		(0.864)
Household saving rate		-0.0839^***^		-0.0824^***^		-0.0809^***^
		(0.000)		(0.000)		(0.000)
GDP per capita		0.792^***^		0.797^***^		0.798^***^
		(0.000)		(0.000)		(0.000)
Occupational fixed effects; Regional fixed effects; Year fixed effects		Yes		Yes		Yes
Constants		-1.902^***^		-1.773^***^		-1.788^***^
		(0.002)		(0.004)		(0.004)
*N*	6882	6882	6882	6882	6882	6882
*R* ^2^	0.026	0.132	0.004	0.134	0.007	0.133
F	101.2	41.83	20.14	40.67	17.05	37.49

Note *, ** and *** indicate significant at the 10%, 5% and 1% levels respectively, with p-values reported in parentheses

### Impact of the commitment of women’s working hours on subjective psychological state

A subjective psychological state is both a subjective reflection of real life and a positive psychological experience of a person’s existence and development. Existing research suggests that subjective psychological state is often bound up with household liabilities and changes in accidents. Therefore, major event expenditures and financial liabilities are used as control variables when subjective psychological state is used as an dependent variable.

The regression results of women’s working hours on subjective psychological state are demonstrated in Table 4. The effects of the duration of women’s working hours, the squared term of working hours and varying degrees of working hours on the household living environment are presented in columns (1)-(9). The estimation results of Oprobit and OLS models are reported in columns (1)-(6) and (7)-(9), respectively.

This can be seen from the estimation results shown in Table 4:

First, the impact trend of women’s working hours on subjective psychological state is positive. The regression coefficients show that, as can be observed, women’s working hours have significantly positive regression coefficients whether subjective welfare is regressed using the Oprobit model as an ordered variable or the OLS model as a continuous variable. To put it another way, the subjective psychological state tends to increase with the increase of women’s working hours. Thus, the increase of working hours is beneficial for subjective psychological status.

Second, women’s working hours do not have a non-linear impact on subjective psychological state, with no phase effects. In terms of the relationship between the two, the primary and secondary terms of women’s working hours are negative but insignificant, and significantly positive, respectively. However, it is possible to calculate that women’s weekly working hours have a probable extreme point of 30 h. In terms of different levels of working hours, with weekly working hours shorter than 30 h as the reference group, the effect of working hours on subjective psychological state is not sensitive in all intervals. Research hypothesis 4 was partially verified. The reason why women’s working hours do not have a phased effect on the subjective psychological state is that the increase in women’s working hours is a “rational choice”, especially for dual-income families, where the female labor force actively extends their working hours. The increase in women’s working hours does not decrease subjective psychological state, and the phased effect is not obvious. The stages are not apparent.

Third, health status, married status and personal income have a positive effect on subjective psychological state. Other control variables also exert an effect on subjective psychological state: Among individual characteristics, good health and marriage are associated with better subjective psychological state. The regression results indicate that the factors of women’s health and marital status have a significant effect on subjective psychological state at the 1% level of significance. The better the health status is, the better the subjective psychological state will be. The results of marital status show that married status contributes to a subjective psychological state. Among work characteristics, personal income has a positive effect on subjective psychological state. The hourly wage of females has a significant effect at the 1% level.

**Table 4:** Impact of women’s working hours on subjective psychological states

	(1)	(2)	(3)	(4)	(5)	(6)	(7)	(8)	(9)
	Oprobit						Robustness tests		
Worktime	0.00354^*^	0.00527^**^	-0.0126^**^	-0.00727			0.0111^***^	-0.00560	
	(0.071)	(0.014)	(0.015)	(0.298)			(0.004)	(0.658)	
Worktime2			0.000125^**^	0.000121^*^				0.000161	
			(0.012)	(0.059)				(0.165)	
Worktime (Less than 30 h = reference group)									
(30,40]					0.0967^**^	0.0473			0.189
					(0.031)	(0.723)			(0.439)
(40,50]					-0.0624	-0.0583			-0.0373
					(0.174)	(0.666)			(0.880)
(50,60]					0.0161	0.000856			0.0447
					(0.727)	(0.995)			(0.856)
(60,max)					0.0187	0.182			0.502^*^
					(0.680)	(0.197)			(0.052)
Age		-0.00831^*^		-0.00837^*^		-0.00868^**^	-0.0142^*^	-0.0143^*^	-0.0152^*^
		(0.058)		(0.056)		(0.048)	(0.074)	(0.072)	(0.057)
Register		0.0505		0.0590		0.0599	0.0211	0.0319	0.0426
		(0.504)		(0.436)		(0.429)	(0.879)	(0.818)	(0.759)
Educ		-0.0935^*^		-0.0749		-0.0829	-0.0722	-0.0474	-0.0553
		(0.067)		(0.150)		(0.111)	(0.440)	(0.618)	(0.561)
Health		0.195^***^		0.193^***^		0.194^***^	0.361^***^	0.359^***^	0.362^***^
		(0.000)		(0.000)		(0.000)	(0.000)	(0.000)	(0.000)
Marriage		0.396^***^		0.391^***^		0.386^***^	0.777^***^	0.769^***^	0.752^***^
		(0.000)		(0.000)		(0.000)	(0.000)	(0.000)	(0.000)
Member		4.014		4.042		4.043	1.471	1.506	1.519
		(0.876)		(0.875)		(0.875)	(0.460)	(0.450)	(0.446)
Lnwage		0.137^***^		0.125^***^		0.112^**^	0.270^***^	0.253^***^	0.231^***^
		(0.002)		(0.005)		(0.012)	(0.001)	(0.002)	(0.005)
Familysize		-0.0109		-0.0139		-0.0151	-0.0238	-0.0275	-0.0327
		(0.543)		(0.440)		(0.407)	(0.472)	(0.407)	(0.329)
Number of children		-0.0761		-0.0705		-0.0708	-0.125	-0.117	-0.117
		(0.177)		(0.212)		(0.210)	(0.222)	(0.253)	(0.256)
Housework		-0.00200		-0.00287		-0.00503	-0.0253	-0.0264	-0.0276
		(0.942)		(0.917)		(0.855)	(0.612)	(0.595)	(0.581)
Household saving rate		0.0402		0.0379		0.0433	0.0569	0.0527	0.0626
		(0.414)		(0.442)		(0.379)	(0.531)	(0.562)	(0.491)
Major event expenditures		-0.00853		-0.00738		-0.00753	-0.00613	-0.00443	-0.00479
		(0.278)		(0.350)		(0.341)	(0.670)	(0.759)	(0.741)
Financial liabilities		0.00316		0.00177		0.00288	0.00783	0.00592	0.00706
		(0.669)		(0.812)		(0.697)	(0.564)	(0.664)	(0.604)
GDP per capita		-0.00881		-0.00210		-0.0306	-0.0347	-0.0257	-0.0862
		(0.952)		(0.988)		(0.835)	(0.896)	(0.923)	(0.747)
Occupational fixed effects; Regional fixed effects; Year fixed effects		Yes		Yes		Yes	Yes	Yes	Yes
Constants							5.726^*^	5.955^**^	6.797^**^
							(0.055)	(0.046)	(0.023)
*N*	1157	1157	1157	1157	1157	1157	1157	1157	1157
*Log likelihood*	-2258.7733	-2063.8971	-2593.5434	-2062.1149	-13121.58	-2063.6591			
*R* ^2^							0.092	0.094	0.094
F							4.411	4.323	4.055

Note *, ** and *** indicate significant at the 10%, 5% and 1% levels respectively, with p-values reported in parentheses

### Analysis of gender differences in the residential effects of working hours

In this section, the impact of working hours on household non-economic welfare was further explored from the perspective of gender. The estimation results using the Oprobit model measure are reported in Table 5. The estimation results of the marginal effects corresponding to regressions are shown in Table 6. As illustrated in Table 6, the dependent variable is the household living environment, and independent variables are the regressions, squared term and segments of working hours to further investigate the impact of the input of working hours on residential effects from a gender perspective.

It can be found from the regression results in Tables 5 and 6 that:

First, the impact of working hours on the household living environment is more significant for females compared to males. As for the significance level of the regression coefficients, working hours have a non-significant effect on the household living environment in the male sample and are significant at the 10% level in the female sample. Regarding the direction of the regression coefficients, both men’s and women’s working hours positively affect the household living environment, and the marginal effects of women’s working hours are positive and higher than those of men’s working hours. Therefore, women’s working hours have a more obvious effect on the household living environment.

Second, the impact of working hours on the household living environment is significantly more inverted “U”-shaped for women than for men, with larger extreme points. Regarding the significance level of the regression coefficients, the coefficients of the primary and secondary terms of working hours are significant at the 10% level for the male sample and 10% or 5% level for the female sample. Regarding the direction and magnitude of the regression coefficients, the coefficients of primary and secondary terms are positive and negative, respectively, in the male sample, and the extreme point is around 64.6 h, which shows an inverted “U”-shaped relationship. The coefficients of primary and secondary terms are positive and negative, respectively, in the female sample, and the extreme point is approximately 68.6 h. Thus, the impact of working hours on the household living environment is inverted “U”-shaped for both males and females.

Third, the impact of the sub-intervals of working hours on the household living environment is more pronounced for females than for males. As regards the significance level of the regression coefficients, the regression coefficients of working hours in all intervals are insignificant in the male sample compared with that of weekly working hours of less than 30 h. Except for 40–50 weekly working hours, working hours in other intervals are significant at the 5% or 1% level in the female sample. Concerning the direction of the regression coefficients, the regression coefficients of working hours in all intervals are positive and negative in the male sample compared with that of weekly working hours less than 30 h, and the regression coefficients of working hours in all intervals are positive in the female sample. Therefore, the effect of the sub-intervals of women’s working hours on the household living environment is more significant in comparison with that of weekly working hours of less than 30 h.

In conclusion, the residential effects of working hours on the household living environment demonstrate clear gender differences. Compared with men’s working hours, women’s working hours more significantly affect the household living environment, and an inverted “U”-shaped relationship can be detected between working hours and the household living environment. Weekly working hours of 30–40, 50–60 and longer than 60 h have a greater impact on the household living environment than those of less than 30 h, while an inverted “U”-shaped relationship can be found between men’s working hours and the household living environment. For men, an inverted U-shaped relationship can be detected between working hours and the household living environment, which is not significant in the aspects of both average working hours and the sub-division of working hours. Thus, women’s working hours more significantly affect housing effects.

**Table 5:** Impact of men’s working hours on the household living environment

	(1)	(2)	(3)
Worktime	0.000860	0.00515^*^	
	(0.256)	(0.051)	
Worktime2		-0.0000399^*^	
		(0.084)	
Worktime(Less than 30 h = reference group)			
(30,40]			0.0385
			(0.446)
(40,50]			-0.00194
			(0.970)
(50,60]			0.0642
			(0.204)
(60,max)			0.0401
			(0.425)
Age	0.00208**	0.00211**	0.00206**
	(0.046)	(0.044)	(0.049)
Educ	0.101***	0.0978***	0.1000***
	(0.000)	(0.000)	(0.000)
Lnwage	0.0995***	0.102***	0.0972***
	(0.000)	(0.000)	(0.000)
Workplace	0.0486	0.0466	0.0466
	(0.113)	(0.128)	(0.132)
Familysize	0.0512***	0.0513***	0.0513***
	(0.000)	(0.000)	(0.000)
Number of children	-0.0374**	-0.0378**	-0.0376**
	(0.024)	(0.022)	(0.023)
Housework	-0.0138	-0.0133	-0.0138
	(0.140)	(0.155)	(0.138)
Financial liabilities	-0.00498*	-0.00484*	-0.00494*
	(0.089)	(0.099)	(0.092)
Household saving rate	-0.0395*	-0.0416*	-0.0393*
	(0.075)	(0.061)	(0.077)
Housing value	0.122***	0.122***	0.123***
	(0.000)	(0.000)	(0.000)
GDP per capita	-0.134***	-0.137***	-0.136***
	(0.010)	(0.009)	(0.009)
Occupational fixed effects; Regional fixed effects; Year fixed effects	Yes	Yes	Yes
N	9159	9159	9159
*Log pseudolikelihood*	-10798.113	-10796.664	-10796.81

Note *, ** and *** indicate significant at the 10%, 5% and 1% levels respectively, with p-values reported in parentheses

**Table 6:** Gender differences in the residential effects of working hours

	marginal effect						
		oprobit_1	oprobit_2	oprobit_3	oprobit_4	oprobit_5	oprobit_6
Worktime	Men	-0.00000688	-0.00000704	-0.000228	-0.0000987	0.000188	0.000153
	women	-0.0000161^*^	-0.0000171^*^	-0.000387^*^	-0.000179^*^	0.000301^*^	0.000298^*^
Worktime extremum points	Men	64.63					
	women	68.78					
Less than 30 h		reference group	reference group	reference group	reference group	reference group	reference group
(30,40]	Men	-0.00031	-0.00032	-0.01022	-0.00443	0.00842	0.0068
	women	-0.00135^***^	-0.00144^***^	-0.0327^***^	-0.01518^***^	0.02546^***^	0.02521^***^
(40,50]	Men	0.000016	0.000016	0.00051	0.00022	-0.00042	-0.00034
	women	-0.00079	-0.00084	-0.019	-0.00884	0.0148	0.0147
(50,60]	Men	-0.00051	-0.00053	-0.017	-0.00737	0.014	0.0114
	women	-0.00172^***^	-0.00183^***^	-0.0415^***^	-0.0193^***^	0.0323^***^	0.0320^***^
(60,max)	Men	-0.00032	-0.00033	-0.0106	-0.00461	0.00877	0.00713
	women	-0.0017^***^	-0.00183^***^	-0.0414^***^	-0.0193^***^	0.0323^***^	0.0320^***^

### Analysis of gender differences in the social effects of working hours

The econometric estimation results using the OLS model are shown in Table 7. In this table, the dependent variable is family social relations, and the independent variables are working hours, the squared term of working hours and the regression results of the segments of working hours to further examine the impact of working hours on social effects from a gender perspective.

It can be seen from the regression results shown in Tables 7 and 8 that:

First, women’s working hours contribute more to family social relations than men’s working hours. Regarding the significance level of the regression coefficients, working hours are significant at the 1% level for male and female samples on family social relations. As regards the direction and size of the regression coefficients, both men’s and women’s working hours positively affect family social relations. The regression coefficient of women’s working hours is 0.00272. This means that for every unit increase in women’s working hours, family social relations improve by about 0.27%, which is higher than the regression coefficient of men’s working hours. Thus, women’s working hours have a more pronounced positive effect on family social relations, which indicates that the entry of women into the labor market is conducive to enhancing social relations like mutual assistance and reciprocity compared with that of men.

Second, women’s working hours have a clear “U”-shaped relationship with family social relations compared to men’s working hours. Regarding the significance level of the regression coefficients, the coefficients of primary and secondary terms of working hours are significant at the 5% level and insignificant, respectively, in the male sample. This indicates that no inverted U-shaped relationship exists between working hours and family social relations in the male sample. However, the coefficients of the primary and secondary terms of working hours are significant at the 1% or 5% level in the female sample, which shows a clear “U”-shaped curve. As regards the direction and magnitude of the regression coefficients, the coefficients of primary and secondary terms are positive and negative, respectively, in the male sample, but fail to constitute a non-linear relationship. However, the coefficients of primary and secondary terms are negative and positive, respectively, in the female sample, with an extreme point of about 35.89 h. Thus, women’s working hours more significantly affect family social relations, with a “U”-shaped relationship.

Third, the effect of the sub-ranges of working hours on family social relations is not significant for females compared to males. Regarding the significance level of the regression coefficients, the regression coefficients of working hours in the male sample are significant at the 10% and 5% levels for 40–50 and 50–60 h per week, respectively, compared with that of weekly working hours less than 30 h. In the female sample, the regression coefficients of working hours are not significant at all intervals. As regards the direction of regression coefficients, the regression coefficients of working hours in all intervals are positive in the male sample and both positive and negative in the female sample compared with that of weekly working hours of less than 30 h. Therefore, the effects of working hours of 40–50 and 50–60 h per week on family social relations are more significant for males than for those working shorter than 30 h per week.

**Table 7:** Impact of men’s working hours on family social relations

	(1)	(2)	(3)
Worktime	0.00196^***^	0.00548^**^	
	(0.004)	(0.019)	
Worktime2		-0.0000328	
		(0.116)	
Worktime(Less than 30 h = reference group)			
(30,40]			0.0383
			(0.387)
(40,50]			0.0763^*^
			(0.085)
(50,60]			0.0866^**^
			(0.048)
(60,max)			0.0701
			(0.116)
Age	0.00286*	0.00291*	0.00285*
	(0.074)	(0.070)	(0.075)
Register	0.0217	0.0208	0.0189
	(0.397)	(0.417)	(0.462)
Educ	0.0195	0.0177	0.0185
	(0.192)	(0.238)	(0.220)
Health	0.0201**	0.0206**	0.0196**
	(0.035)	(0.031)	(0.040)
Marriage	0.168***	0.167***	0.170***
	(0.000)	(0.000)	(0.000)
Member	0.233***	0.235***	0.237***
	(0.000)	(0.000)	(0.000)
Lnwage	0.144***	0.146***	0.136***
	(0.000)	(0.000)	(0.000)
Workplace	0.157***	0.156***	0.156***
	(0.000)	(0.000)	(0.000)
Number of children	-0.109***	-0.109***	-0.107***
	(0.000)	(0.000)	(0.000)
Number of old	-0.0784***	-0.0786***	-0.0789***
	(0.000)	(0.000)	(0.000)
Housework	0.0141	0.0146	0.0139
	(0.150)	(0.134)	(0.156)
Household saving rate	-0.186***	-0.187***	-0.186***
	(0.000)	(0.000)	(0.000)
GDP per capita	0.681***	0.683***	0.683***
	(0.000)	(0.000)	(0.000)
Occupational fixed effects; Regional fixed effects; Year fixed effects	Yes	Yes	Yes
Constants	-0.235	-0.337	-0.186
	(0.637)	(0.502)	(0.708)
*N*	10,104	10,104	10,104
*R* ^2^	0.116	0.116	0.116
F	52.98	51.04	47.15

Note *, ** and *** indicate significant at the 10%, 5% and 1% levels respectively, with p-values reported in parentheses

**Table 8:** Gender differences in the social effects of working hours

		Worktime	Working hours extremum points	Less than 30 h	(30,40]	(40,50]	(50,60]	(60,max)
family social relations(%)	Men	0.20^***^	—	reference group	3.90	7.93^*^	9.05^**^	7.26
	Women	0.27^***^	35.89	reference group	3.19	-6.81	0.93	7.71

The effects corresponding to household consumption expenditures and per capita household income in the table are exponentially converted, exp()-1

To sum up, the social effects of working hours on family social relations show clear gender differences. Compared with men’s working hours, women’s working hours have a more significant effect on family social relations, and a “U”-shaped relationship can be found between working hours and family social relations. Men’s working hours more significantly affect family social relations. Compared with less than 30 h of weekly working hours, 50–60 h of weekly working hours have a higher degree of effect on family social relations. Therefore, women’s working hours, the squared term of women’s working hours and the sub-areas of men’s working hours have more significant effects on social relations.

### Analysis of gender differences in the psychological effects of the investment of working hours

The econometric estimation results using the Oprobit model are reported in Table 9. In this table, the dependent variable is the subjective psychological state, and independent variables are the regression results, squared term and segments of working hours to further examine the impact of working hours on psychological effects from the perspective of gender.

It can be seen from the regression results in Tables 9 and 10 that:

First, the impact of working hours on subjective psychological state is more significant for females compared to males. Regarding the significance level of the regression coefficients, working hours are significant at the 5% level for both males and females. As for the direction and marginal effects of the regression coefficients, the effect of working hours on the subjective psychological state from low to high is shown from positive to negative in the male sample, while that on the subjective psychological state from low to high is shown from negative to positive in the female sample. Therefore, the overall effect of working hours on subjective psychological state is positive for females and negative for males.

Second, no non-linear relationship exists between working hours and subjective psychological state for either males or females. Regarding the significance level of the regression coefficients, the coefficients of the primary and secondary terms of working hours are significant at the 10% level and insignificant in the male sample. Similarly, the coefficients of the primary and secondary terms of working hours are insignificant and significant at the 10% level in the female sample. As regards the direction and magnitude of the regression coefficients, the coefficients of primary and secondary terms are negative and positive, respectively, in the male sample, but do not form a “U”-shaped curve. Nevertheless, the coefficients of primary and secondary terms are negative and positive, respectively, in the female sample, and do not form a “U”-shaped curve either. Thus, no non-linear relationship can be detected between working hours and subjective psychological state for men and women.

Third, the impact of the sub-intervals of working hours on subjective psychological states is insensitive for females compared to males. Regarding the significance level of the regression coefficients, the regression coefficients of working hours at 30–40 and 40–50 h per week in the male sample are significant at 5% and 1% levels, respectively, compared with that of weekly working hours shorter than 30 h. However, the regression coefficients of working hours at each interval in the female sample are not significant. As regards the direction and marginal effects of the regression coefficients, the regression coefficients of working hours in all intervals are negative in the male sample compared with that of weekly working hours shorter than 30 h, with stronger negative effects in the weekly working hours of 30–40 and 40–50 h. The regression coefficients of working hours in all intervals in the female sample are both positive and negative. Thus, the effect of the sub-intervals of working hours on subjective psychological state is more significant for males compared with that of weekly working hours of less than 30 h.

To conclude, the psychological effects of working hours on subjective mental states show significant gender differences. Compared with men’s working hours, women’s working hours positively affect psychological effects, and neither men nor women have a non-linear relationship with psychological effects. Men’s weekly working hours of 30–40 and 40–50 h have a stronger effect on psychological effects than those of less than 30 h.

**Table 9:** Effect of the commitment of working hours on subjective psychological states in men

	(1)	(2)	(3)
Worktime	-0.00327^**^	-0.0104^*^	
	(0.045)	(0.074)	
Worktime2		0.0000662	
		(0.203)	
Worktime(Less than 30 h = reference group)			
(30,40]			-0.220^**^
			(0.036)
(40,50]			-0.335^***^
			(0.001)
(50,60]			-0.149
			(0.149)
(60,max)			-0.122
			(0.246)
Age	-0.0146***	-0.0145***	-0.0148***
	(0.000)	(0.000)	(0.000)
Register	0.120**	0.124**	0.133**
	(0.050)	(0.042)	(0.031)
Educ	-0.0554	-0.0505	-0.0348
	(0.135)	(0.176)	(0.354)
Health	0.228***	0.229***	0.230***
	(0.000)	(0.000)	(0.000)
Marriage	0.535***	0.530***	0.535***
	(0.000)	(0.000)	(0.000)
Member	0.0901	0.113	0.177
	(0.815)	(0.769)	(0.646)
Lnwage	-0.0592*	-0.0621*	-0.0286
	(0.076)	(0.063)	(0.383)
Familysize	0.0502***	0.0512***	0.0504***
	(0.000)	(0.000)	(0.000)
Number of children	-0.142***	-0.143***	-0.152***
	(0.001)	(0.001)	(0.000)
Housework	-0.0117	-0.0126	-0.0103
	(0.636)	(0.609)	(0.677)
Household saving rate	-0.0229	-0.0225	-0.0232
	(0.577)	(0.584)	(0.572)
Major event expenditures	0.00844	0.00955	0.0111*
	(0.197)	(0.148)	(0.094)
Financial liabilities	-0.00518	-0.00575	-0.00683
	(0.363)	(0.314)	(0.233)
GDP per capita	-0.0789	-0.0796	-0.0492
	(0.509)	(0.505)	(0.683)
Occupational fixed effects; Regional fixed effects; Year fixed effects	Yes	Yes	Yes
N	1817	1817	1817
*Log likelihood*	-3292.9452	-3292.1341	-3288.1636

**Table 10:** Gender differences in the psychological effects of working hours

	marginal effect						
		oprobit_1	oprobit_2	oprobit_3	oprobit_4	oprobit_5	oprobit_6
Worktime	Men	0.0000246	0.0000131	0.0000316^*^	0.0000915^*^	0.000116^*^	0.000491^**^
	Women	-0.000117^**^	-0.0000318	-0.0000455^*^	-0.0000931^**^	-0.000162^**^	-0.000737^**^
Less than 30 h		reference group	reference group	reference group	reference group	reference group	reference group
(30,40]	Men	0.0016^*^	0.0009	0.0021^*^	0.0062^**^	0.0078^**^	0.0328^**^
	Women	-0.0010	-0.0003	-0.0004	-0.0008	-0.0015	-0.0066
(40,50]	Men	0.00243^**^	0.00131^*^	0.00322^**^	0.00940^***^	0.0119^***^	0.0500^***^
	Women	0.00127	0.00035	0.000501	0.00103	0.0018	0.00819
(50,60]	Men	0.00108	0.000584	0.00143	0.00418	0.00529	0.0223
	Women	-0.0000187	-0.00000513	-0.00000736	-0.0000151	-0.0000264	-0.00012
(60,max)	Men	0.000884	0.000479	0.00117	0.00343	0.00433	0.0182
	Women	-0.00397	-0.00109	-0.00156	-0.00321	-0.00561	-0.0255
		oprobit_7	oprobit_8	oprobit_9	oprobit_10	oprobit_11	
Worktime	Men	0.000292^**^	0.000158^**^	0.00000581	-0.000243^**^	-0.000982^**^	
	Women	-0.000379^**^	-0.000269^**^	-0.000143	0.000436^**^	0.00154^**^	
Less than 30 h		reference group	reference group	reference group	reference group	reference group	
(30,40]	Men	0.0196^**^	0.0107^**^	0.0005	− 0.0164^**^	-0.0657^**^	
	Women	-0.0034	-0.0024	-0.0013	0.0039	0.0138	
(40,50]	Men	0.0299^***^	0.0163^***^	0.000707	-0.0250^***^	-0.100^***^	
	Women	0.00419	0.00297	0.00157	-0.00481	-0.0171	
(50,60]	Men	0.0133	0.00724	0.000315	-0.0111	-0.0445	
	Women	-0.0000615	-0.0000436	-0.000023	0.0000706	0.00025	
(60,max)	Men	0.0109	0.00593	0.000258	-0.00913	-0.0365	
	Women	-0.0131	-0.00925	-0.00489	0.015	0.0532	

Note There are no extreme points for both male and women working hourss, and the marginal effects of the squared working hours term are not shown in the table

## Research conclusions and policy implications

### Research conclusions

First, in terms of household non-economic welfare, women’s working hours have a greater impact on residential and psychological effects. In this study, it was found that working hours divided into continuous, squared term and sub-interval analyses have different impacts on residential, social and psychological effects, respectively. On average, the psychological effects of women’s working hours are the largest, with an average increase of one hour per week boosting the psychological effects by 0.53%. The effects of women’s working hours on residential and social effects have extreme points, which are 68.78 and 35.89 h, respectively, and both statistically and practically significant. The social effects of women’s working hours show a “U”-shaped curve. When weekly working hours exceed 35.89 h, it promotes an increase in social effects. The residential effects of women’s working hours exhibit an inverted “U”-shaped curve. When weekly working hours are shorter than 68.78 h, it promotes an increase in residential effects. In terms of intervals, differences exist in the impacts of different intervals of working hours on residential effects. Women’s working hours have a residential effect of 12.7–16.2% when compared with weekly working hours less than 30 h, and women’s weekly working hours exceeding 60 h have the greatest impact on residential effects at 16.2%.

Second, in terms of residential effects, the extreme point of maximizing the residential effects of the investment of women’s working hours is 68.78 h. Compared with weekly working hours of less than 30 h, the residential effects of the investment of women’s working hours are higher than those of men’s working hours. In terms of social effects, the extreme point of minimizing family social relations for women is 35.89 h. Compared with weekly working hours of less than 30 h, the social effects of the investment of women’s working hours are weaker than those of men’s working hours. Concerning psychological effects, women’s working hours positively affect psychological effects, while men’s working hours negatively influence psychological effects. Compared with weekly working hours of less than 30 h, women’s working hours have a lower degree of influence on subjective psychological state than men’s working hours.

### Policy discussions

Firstly, the system of working hours and working arrangements should be improved to create a family-friendly working environment. The best policy orientation is to help women play the dual role of working women who have their careers and can become mothers through public policies. In terms of labor participation, it is important to foster a social atmosphere conducive to the participation of women in the workplace and bind them to traditional gender identity norms. By this means, women can actively get involved in the labor market and fully exploit the benefits of female employment for families and society. Concerning working hours, based on the current systems of maternity, paternity and family care leaves, a flexible working hour system should be established to shorten the daily or weekly working hours of workers with family responsibilities, to satisfy the childcare needs of workers and the care of other close family members. As for work systems, enterprises are incentivized to employ flexible work systems and flexible work arrangements to effectively support women in striking a balance between childcare and work. In particular, it is important to note that disciplinary measures should not be formulated to target enterprises. Instead, it is necessary to support enterprises from the perspective of their aspirations, to achieve work-family balance for women through support for enterprises. In terms of public services, it is critical to redistribute unpaid care services between the sexes through family-friendly employment measures instead of constructing employment measures only friendly to male workers or some female workers. From the angle of workers, particular work objectives should be clarified in practice to raise labor efficiency, enhance labor value, reduce labor involution and improve the sense of welfare.

Secondly, the sustainable development of families should be promoted in terms of their functions and needs. Based on general policy arrangements to enhance the development capacity of families, there is a need to provide targeted support for families in light of changing trends in families and the weakening of family functions. At the same time, it is necessary to enhance the development capacity of families through special policies to help families in difficulty, like those that remain unable to maintain basic survival through labor supply. The family development policy of the government is not a substitute for the basic functions of families per se but aims to support and strengthen the functions of families based on families giving full play to their potential.

## Future research and limitations

1. Limitations of this study. In this study, the focus was on the impact of working hours on households, and research on working hours was combined with the relationship, trends and extent of household non-economic welfare. In the future, more influences could be studied, including the relationship between housework time and household non-economic welfare, gender differences, etc.
2. Future research potential.From the data side, international comparisons and cross-corroboration can be performed using micro-databases from different countries to more accurately explore the impact of working hours on household non-economic welfare. From the research subject. Notably, the subject of the study, namely, the impact of women’s working hours on household non-economic welfare, and gender differences, can be used for testing the validity of the model in other fields. Further studies could be carried out in the future to determine whether different levels of education, age groups, marital statuses or family structures are consistent with these study findings.

## Data Availability

The datasets used during the current study are available from website of Institute of Social Science Survey(https://isss.pku.edu.cn/cfps/download/login).

## References

[1] George NM, Parida V, Lahti T, Wincent J. A systematic literature review of entrepreneurial opportunity recognition: insights on influencing factors. Int Entrepreneurship Manage J. 2016;12(2):309–50.

[2] Chu LM, Zhang Q. Do women’s working hours inputs yield higher household economic welfare than men’s? Heliyon. 2023;9(11):1–11. 10.1016/j.heliyon.2023.e21437 PMC10643254 38027939

[3] Brosch EK, Binnewies C. A diary study on predictors of the work-life interface: the role of time pressure, psychological climate and positive affective states. Manage Revue. 2018;29(1):55–78.

[4] Liu ZY. The choice of family welfare policies at the present stage in China–thinking based on improving the development capacity of families. J Party Administrative Cadres. 2011;8:55–9.

[5] Assessment ME. Ecosystems and human well-being scenarios: findings of the scenarios working group. Covelo: Island; 2005.

[6] Bullinger C. Comparison of local government’s policies on Kutai and Dayak Benuaq villages in Kutai Barat, Indonesia: factors influencing village life and household well-being since decentralization. Bogor, Indonesia: Center for International Forestry Research; 2006. p. 35.

[7] Song K, Xu Y. Does digital finance improve the welfare of rural households? Based on Sen’s feasibility theory. World Agric. 2023;4:61–70.

[8] Haushofer J, Fehr E. On the psychology of poverty. Science. 2014;344:862–7. 10.1126/science.1232491 24855262

[9] Albuquerque B, Krustev G. Debt overhang and deleveraging in the US household sector: gauging the impact on consumption. Rev Income Wealth. 2018;2:459–81.

[10] Wang D, Qian W, Guo X. Gains and losses: does farmland acquisition harm farmers’ welfare? Land Use Policy. 2019;86:78–90.

[11] Alkire S, Santos ME. Acute multidimensional poverty: a new index for developing countries. OPHI Working Papers 38, Oxford Poverty and Human Development Initiative (OPHI), University of Oxford; 2011.

[12] Li H, Zhang XL, Li H. Has farmer welfare improved after rural residential land circulation? J Rural Stud. 2022;93(7):479–86.

[13] Xiao W, Wu M. Life-cycle factors and entrepreneurship: evidence from rural China. Small Bus Econ. 2021;57:2017–40.

[14] Williams DT, Baker RS. Family structure, risks, and racial stratification in poverty. Soc Probl. 2021;68(4):964–85.

[15] Su JF, Guo SRH. Capital and rural households’ vulnerability to relative poverty: evidence from China. Discrete Dynamics Nat Soc. 2022;5:1–11.

[16] Huang L, Huang SG. Digital finance, resident labor participation and household financial vulnerability. J Technol Econ. 2023;12:109–24.

[17] Feng J, Huang W, Lyu S. How do Chinese households respond to hospitalization? Evidence from monthly panel data. J Dev Econ. 2021;150:1–21.

[18] Fadlon I, Nielsen TH. Family labor supply responses to severe health shocks: evidence from Danish administrative record. Am Economic J Appl Econ. 2021;13(3):1–30.

[19] Clark AE, Frijters P, Michael ASR. Income, happiness, and urility: explanation for the Easterlin paradox and other puzzles. J Econ Lit. 2008;46:95–144.

[20] Zhu HG, Fu YZ. Labor employment and family welfare effect in key state-owned forest areas. J Agro-Forestry Econ Manage. 2020;2:190–9.

[21] Yang HL, Hu HYD. Will delayed retirement occupy family fertility? J Financ Econ. 2018;44(10):53–66.

[22] Cheng Q, Zheng YF, Xu JX. Family endowment, structural constraints and labor supply of married women: evidence from Chinese general social survey of 2010. Stud Labor Econ. 2017;5(2):80–95.

[23] Zhao CY. Demographic dividend, structural bonus and regional economic growth. Northwest Population J. 2018;39(6):23–31.

[24] Maslow A H. A theory of human motivation. Psychol Rev. 1943;50(4):370–96.

[25] Sen A. Capability and well-being. Qual Life. 1993;25(6):270–93.

[26] Sen A. Capitalism beyond the crisis. New York Rev Books. 2009;56(5):26–35.

[27] Bin HG. Spending and urban household consumption—an empirical study based on status seeking. Stat Res. 2015;4:68–76.

